# Transforming growth factor-β2 increases extracellular matrix proteins in optic nerve head cells via activation of the Smad signaling pathway

**Published:** 2011-06-28

**Authors:** Gulab S. Zode, Anirudh Sethi, Anne-Marie Brun-Zinkernagel, I-Fen Chang, Abbot F. Clark, Robert J. Wordinger

**Affiliations:** Department of Cell Biology and Genetics, University of North Texas Health Science Center at Fort Worth, Fort Worth, TX

## Abstract

**Purpose:**

Transforming growth factor-β2 (TGF-β2) is associated with glaucomatous neuropathy, primarily via the increased synthesis and secretion of extracellular matrix (ECM) proteins and remodeling of the optic nerve head (ONH). Here, we investigated the signaling pathways used by TGF-β2 to stimulate ECM expression by ONH astrocytes and lamina cribrosa (LC) cells.

**Methods:**

TGF-β2 localization and secretion was examined in human donor tissues and ONH astrocytes and LC cells. To examine TGF-β2 signaling, ONH astrocytes and LC cells were treated with recombinant TGF-β2, and phosphorylation of Smad and non-Smad signaling proteins were examined by western blot analyses and immunostaining.

**Results:**

TGF-β2 is significantly increased in the LC region of the ONH in glaucomatous eyes compared to age-matched normal eyes (n=4, p<0.0013). ONH astrocytes and LC cells secrete TGF-β2, indicating that these cells may be an in vivo source of TGF-β2 in the human ONH. In addition, treatment of ONH astrocytes and LC cells with exogenous TGF-β2 increased ECM protein synthesis and secretion. With respect to TGF-β2 signaling, recombinant TGF-β2 induced phosphorylation of canonical signaling proteins Smad2/3 but did not alter phosphorylation of non-canonical signaling proteins extracellular signal-regulated kinases (ERK)1/2, p38, and c-Jun N-terminal kinases (JNK)1/2 proteins in ONH astrocytes and LC cells. Exogenous TGF-β2 increased co-localization of pSmad2/3 with Co-Smad4 in the nucleus of ONH astrocytes and LC cells further indicating activation of the canonical Smad signaling pathway. Furthermore, inhibition of TGF-β I receptor activity by SB431542 or inhibition of Smad3 phosphorylation by SIS3 blocked TGF-β2 stimulated ECM expression as well as activation of downstream canonical pathway signaling molecules. Knockdown of either Smad2 or Smad3 via small interfering RNA (siRNA) reduced TGF-β2 stimulated ECM proteins in ONH astrocytes and LC cells.

**Conclusions:**

These studies indicate that TGF-β2 utilizes the canonical Smad signaling pathway to stimulate ECM synthesis in human ONH cells. Our studies also indicate that pSmad2/3 is required for TGF-β2 stimulation of ECM remodeling.

## Introduction

Primary open angle glaucoma (POAG) is a progressive optic neuropathy, characterized by the irreversible loss of retinal ganglion cell (RGC) axons [1]. The pathogenic factors responsible for POAG are still unknown. However, elevated intraocular pressure (IOP) is a major causative and treatable risk factor [2,3]. Chronic elevation of IOP induces optic nerve head (ONH) changes [4,5], including compression of retinal ganglion cell axons at the level of the lamina cribrosa (LC), blockage of axoplasmic flow, and inhibition of retrograde neurotrophin transport to RGC [6-8]. The glaucomatous ONH shows characteristic cupping and excavation of the optic disc, collapse and remodeling of the LC, and activation of ONH astrocytes [4,9,10].

The LC region of the ONH consists of a characteristic sieve-like structure through which RGC axons exit the eye [7,11]. These laminar plates contain extracellular matrix proteins such as elastin and collagens (I, III, V, and VI) [12]. Correct organization and assembly of the collagen and elastin fibers in the LC provides both a supportive framework and elasticity to the ONH, which is believed to protect RGC axons from mechanical stress [13,14]. Major cell types present in the human ONH include ONH astrocytes and LC cells [15,16]. These cells support RGC axons by synthesizing growth factors (e.g., neurotrophins) and extracellular matrix (ECM) proteins [16-19].

Remodeling of the ECM, including changes in fibrillar collagens, basement membrane components, and elastin composition, is characteristic of the glaucomatous ONH [20-23]. The extracellular matrix (ECM) changes include backward bowing of the laminar plates with increased amounts of collagen I, IV, and VI. Altered elastin deposition in LC is thought to alter the elastic properties of the ONH [24]. Increased synthesis and deposition of ECM proteins in the LC region may disrupt nutritional and mechanical support to RGC axons, resulting in RGC atrophy. Several studies suggest that ONH astrocytes and LC cells respond to elevated IOP by increasing transforming growth factor-β2 (TGF-β2) synthesis in the LC region [25-27], which in turn causes altered ECM protein expression.

TGF-β2 belongs to the TGF-β superfamily and plays a fundamental role in the biology of the ECM [28]. In fibrotic diseases, elevated TGF-β2 levels lead to the pathological deposition of ECM proteins [29,30]. TGF-β2 appears to be involved in the pathogenesis of POAG. Patients with glaucoma have higher levels of TGF-β2 in their aqueous humor [31], and TGF-β2 has been shown to increase ECM protein in human trabecular meshwork (TM) cells [32-34]. In addition, TGF-β2 increased IOP in cultured human perfused-anterior eye segments [32,35]. Furthermore, adenoviral gene transfer of active TGF-β2 elevates IOP in mice and rats and reduces outflow facility in mice [36]. Robertson et al. [37] also reported that gene transfer of TGF-β1 into the anterior chamber of rats elevated IOP.

A similar pathophysiology is observed in glaucomatous ONH including elevated TGF-β2 and increased deposition of ECM proteins. In the glaucomatous ONH, elevated TGF-β2 is associated with ECM remodeling [38]. Fuchshofer and colleagues demonstrated that TGF-β2 treatment of cultured ONH astrocytes upregulates mRNA and protein expression of collagen I, collagen IV, fibronectin, connective tissues growth factor (CTGF), tissue transglutaminase (TGM2), and thrombospondin-1 (TSP-1) [17]. These observations suggest that TGF-β2 could be an initiation factor in ECM remodeling in the glaucomatous ONH.

TGF-β2 signaling involves ligand binding to TGF-β receptors and activation of the canonical downstream Smad signaling pathway or non-Smad signaling pathways [39,40]. TGF-β2 dimers bind to the type II receptor, which transphosphorylates the type I receptor. In the canonical Smad signaling pathway, the activated type I receptor then phosphorylates Smads (Smad2/3), which triggers heterodimerization with Co-Smad4 and translocation of the complex to the nucleus to activate specific gene targets. In non-Smad signaling pathways, activated TGF-β receptors utilize extracellular signal-regulated kinases (ERK), P38 mitogen-activated protein kinases (p38^MAPK^), or c-Jun N-terminal kinases (JNK) signaling proteins to activate gene targets [41]. Although TGF-β may utilize ERK, p38^MAPK^, or JNK signaling pathways in various cell types, Smad 2 and Smad3 are thought to be primary TGF-β2-driven fibrogenesis signals in many cell types, including mesangial cells, retinal pigment epithelial cells, and skin fibroblasts [29,42].

Little is known about the underlying signaling mechanisms responsible for TGF-β2 mediated synthesis and deposition of ECM proteins in the normal or glaucomatous ONH. We have previously reported that bone morphogenetic protein-4 (BMP-4) and Smad signaling proteins are present in human ONH tissues, isolated ONH astrocytes, and LC cells and that exogenous BMP-4 treatment of isolated ONH cells resulted in activation of the canonical signaling pathway [18]. However it is not clear if the TGF-β2 canonical Smad signaling pathway or non-Smad signaling pathways are used to regulate ECM protein synthesis and secretion in ONH astrocytes and/or LC cells. In this study, we examined TGF-β2 stimulation of ECM synthesis and deposition in isolated human ONH astrocytes and LC cells to determine whether canonical or non-canonical signaling pathways are used.

## Materials

### Optic nerve head dissection and cell culture

Human ONH astrocytes and LC cells were generated from dissected ONHs and characterized according to previous reports [16,43]. Briefly, human donor eyes from regional eye banks were obtained within 24 h of death, and the LC region of the ONH was dissected from the remaining ocular tissue. The LC tissues were cut into three to four explants and placed in culture plates containing Dulbecco’s modified Eagle’s medium (DMEM, HyClone Laboratories, Logan, UT) containing L-glutamine (0.292 mg/ml, Gibco BRL Life Technologies, Grand Island, NY), penicillin (100 units/ml,)/streptomycin (0.1 mg/ml, Gibco BRL Life Technologies), amphotericin B (4 μg/ml; Gibco BRL Life Technologies), and 10% fetal bovine serum (Gibco BRL Life Technologies).

### Treatment of ONH astrocytes and lamina cribrosa cells

ONH astrocytes and LC cells were grown in 12 well plates. Confluent cells were washed twice with a sterile phosphate buffer solution (PBS), and were kept in serum-free DMEM for 24 h. A fresh serum-free medium with recombinant TGF-β2 was added to ONH astrocytes and LC cells. For the TGF-β2 dose–response study, ONH astrocytes and LC cells were incubated with various concentrations of TGF-β2 (1.25, 2.5, 5, 10, and 20 ng/ml) for 24 h. ONH astrocytes and LC cells grown in the serum-free medium for 24 h were considered to be the control for the above experiment. Cell lysates and the culture medium were collected and analyzed for ECM proteins.

For phosphorylation studies of Smads, ERK1/2, p38, or JNK1/2, confluent ONH astrocytes and LC cells were washed twice with PBS and kept in a serum-free medium for 24 h. A fresh serum-free medium was then added and the cells were incubated with recombinant TGF-β2 (5 ng/ml) for 30 min, 60 min, or 120 min. Cell lysates were collected and immunoblotting with phospho-specific antibodies was used to analyze the various signaling pathways.

To examine the effect of inhibiting the type I TGF-β receptor (Alk5) or inhibiting Smad3 phosphorylation, the cells were pre-incubated with SB431542 (10 uM, Pro. #S4317; Sigma-Aldrich, St. Louis, MO) or SIS3 (25 um and 50 uM; EMD Chemicals, Inc., San Diego, CA), respectively, for 1 h before treatment. The ONH astrocytes and LC cells were incubated with recombinant TGF-β2 for 24 h, with and without SB431542 or SIS3, and cell lysates and the conditioned medium were analyzed for their effects on ECM proteins.

To examine the effect of SB431542 or SIS3 on Smad and non-Smad signaling pathways, ONH astrocytes and LC cells were pre-incubated with SB431542 or SIS3 for 1 h and were then treated with TGF-β2 for 1 h. Cell lysates were then subjected to an analysis of the phosphorylation of Smad and non-Smad signaling molecules by western immunoblotting.

### Immunohistochemistry

Four sets of age-matched normal (76, 79, 84, and 97 years of age) and glaucomatous (72, 78, 82, and 99 years of age) human eyes were obtained from regional eye banks within 6 h of death and were fixed in 10% formalin. Fixed tissues were dehydrated, embedded in paraffin, and 8 um sections were obtained. Sections were deparaffinized, rehydrated, and placed in 0.1% triton, followed by 20 mM glycine for 15 min each. Sections were blocked in 10% normal serum. Slides were incubated overnight with a primary antibody (Table 1) diluted 1:100 in 1.5% (v/v) normal serum, were washed three times with PBS and this was followed by a 2 h incubation in appropriate Alexa Fluor™ secondary antibodies (1:200; Invitrogen Corporation, Carlsbad, CA). Sections were subsequently incubated with DAPI for 30 min to stain the nuclei, and were then washed and mounted. Images were captured using a Zeiss 410 confocal imaging system (Carl Zeiss, Thornwood, NY).

**Table 1:** List of antibodies

**Antibody (cat #), dilutions, source**
Fibronectin (sc-18827), 1:1000, Santa Cruz Biotechnology, Inc. Santa Cruz, CA.
PAI-1 (sc-5297), 1:1000, Santa Cruz Biotechnology, Inc. Santa Cruz, CA.
TGF-β2 (sc-90), 1:500, Santa Cruz Biotechnology, Inc. Santa Cruz, CA.
Collagen I (sc-8783), 1:500, Santa Cruz Biotechnology, Inc. Santa Cruz, CA.
Collagen VI (AB-7821), 1:2000, Millipore Corporate, Billerica, MA
Elastin (MAB-2503), 1:1000, Millipore Corporate, Billerica, MA
β-actin (MAB-1501), 1:2000, Millipore Corporate, Billerica, MA
Goat Anti Mouse, 1:20000, Santa Cruz Biotechnology, Inc. Santa Cruz, CA.
Goat Anti Rabbit, 1: 20000, Santa Cruz Biotechnology, Inc. Santa Cruz, CA.
pSmad3 (#9520), 1:500, Cell Signaling Technology. Inc. Danvers, MA
Smad3 (#9513), 1:500, Cell Signaling Technology. Inc. Danvers, MA
Pp38MAPK (#9215), 1:500, Cell Signaling Technology. Inc. Danvers, MA
P38MAPK (#9212), 1:500, Cell Signaling Technology. Inc. Danvers, MA
pJNK1/2 (#4668), 1:500, Cell Signaling Technology. Inc. Danvers, MA
JNK1/2 (#9252), 1:500, Cell Signaling Technology. Inc. Danvers, MA
pERK1/2 (sc-81492), 1:500, Santa Cruz Biotechnology, Inc. Santa Cruz, CA.
ERK1/2 (#4695), 1:500, Cell Signaling Technology. Inc. Danvers, MA
pSmad2 (#3104), 1:500, Cell Signaling Technology. Inc. Danvers, MA
Smad2 (#3122), 1:500, Cell Signaling Technology. Inc. Danvers, MA

**Figure 1:**
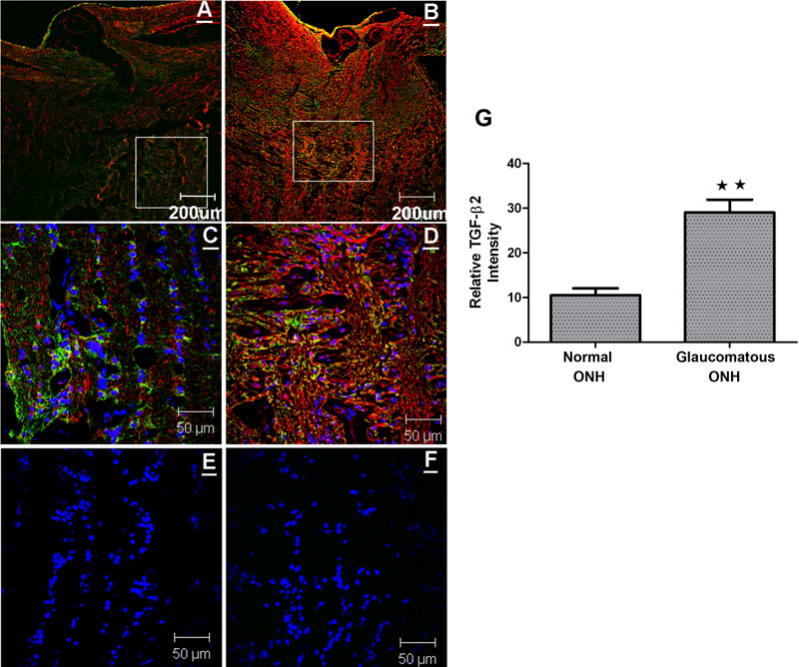
Immunohistochemical evaluation of transforming growth factor (TGF)-β2 expression in normal and glaucomatous optic nerve head (ONH) tissues. A representative immunostaining for TGF-β2 expression in age-matched normal (97 years) and glaucomatous (99 years) human ONH tissues. **A**: ONH tissue section from a normal human donor stained for TGF-β2 (red), glial fibrillary acidic protein (GFAP; green), and 4',6-diamidino-2-phenylindole (DAPI; blue) at lower magnification, and **C**: showing part of the lamina cribrosa (LC) in the white box, at higher magnification. **B**: ONH tissue section from glaucomatous human eyes stained for TGF-β2 (red), GFAP (green), and DAPI (blue) at lower magnification, and **D**: showing LC in the white box, at higher magnification. **E**: Control (no primary antibody for TGF-β2 and GFAP) merged with DAPI in normal ONH, and **F**: in glaucomatous ONH. **G**: Relative intensity measurements of TGF-β2 staining in the LC region in four age-matched normal and glaucomatous ONH tissues, showing significant higher staining for TGF-β2 in glaucomatous ONH tissues compared to normal human ONH tissues [student’s *t*-test, p<0.0013; ** versus normal human ONH tissues; n=4].

For orientation purposes, low power (10×) magnification images were taken, which demonstrated ONH, retina, LC, and blood vessels. For detailed study of TGF-β2 staining in the LC, higher power magnification images were taken within the LC region (white box in Figure 1). The relative intensity of TGF-β2 staining was analyzed using ImageJ software (NIH) with the RBG split option, thereby deconvoluting the green, red, and blue channels. Subsequently, the red channel (for TGF-β2) was selected and the relative intensity was measured. The area for each image was kept constant since the entire red channel was selected. A statistical analysis of the staining intensity was performed with an unpaired Student’s *t*-test using GraphPadPrism 5 (La Jolla, CA).

### Co-localization of pSmad3 and Smad4

Lamina cribrosa cells were grown on glass coverslips. Confluent cells were kept in a serum-free medium for 24 h and were incubated with recombinant TGF-β2 (5 ng/ml) in a serum-free medium for 60 min. The cells were then fixed with 3.5% formaldehyde (in PBS) for 10 min. The coverslips were washed with PBS and treated with 0.02% triton for 10 min. The coverslips were washed twice with PBS and blocked with 10% normal donkey serum for 1 h. The coverslips were then incubated overnight with primary antibodies for pSmad3 or pSmad2 and Co-Smad4 (Table 1), were washed 4 times with PBS, and were incubated with secondary antibodies for 2 h. The specimens were then incubated with DAPI for 30 min to stain the nuclei, and were subsequently washed and mounted. Images were captured using a Zeiss 410 confocal imaging system (Carl Zeiss).

### Protein extraction and western blot analysis

#### Cell lysate

Total cellular protein was extracted from cultured ONH astrocytes and LC cells using a Mammalian Protein Extraction Buffer (#78501; Pierce Biotech, Rockford, IL) with a Protease Inhibitor Cocktail (#78415; Pierce Biotech). Protein concentration was determined using the Bio-Rad Dc protein assay system (Bio-Rad Laboratories, Richmond, CA). Cellular proteins were separated on denaturing polyacrylamide gels and then transferred to PVDF membranes by electrophoresis. Blots were blocked with SuperBlock Blocking Buffer (Prod# 37537; Pierce Biotech) for 1 h. The blots were then incubated overnight with specific primary antibodies (Table 1). The membranes were washed with Tris-buffered saline/Tween buffer (TBST) and were incubated with a corresponding horseradish peroxidase-conjugated secondary antibody (Table 1). The proteins were then visualized in a Fluor Chem^TM^ 8900 imager (Alpha Innotech Corporation, San Leandro, CA) using ECL detection reagents (SuperSignal West Femto Maximum Sensitivity Substrate; Pierce Biotechnology). To ensure equal protein loading, the same blot was subsequently incubated with a β-actin monoclonal antibody and the blot was developed using a horseradish peroxidase-conjugated secondary antibody.

#### Conditioned medium

To detect secreted TGF-β2 proteins in the conditioned medium, confluent ONH astrocytes and LC cells were grown in a serum-free medium for 24 h. The culture medium was then concentrated 20 times and equal volumes of the conditioned medium were analyzed via western immunoblotting, as described above.

### ELISA immunoassay for fibronectin

The conditioned mediums obtained from three ONH astrocytes cell lines and three LC cell lines were centrifuged at 2000 rpm to remove cellular debris. A total 50 ul of the conditioned medium was diluted to 150 ul with a dilution buffer, and soluble fibronectin was quantified using a commercially available enzyme-linked immunosorbent assay (ELISA) kit (cat # ECM 300; Chemicon International, Temecula, CA). The amounts of soluble fibronectin (ug/ml) were plotted for each treatment using GraphPadPrism 5.

### Small interfering RNA and transfection

Small interfering RNA (siRNA) for *Smad3* and non-targeting siRNA controls were purchased from Dharmacon (SMARTpool, La Jolla, CA). Transfection of siRNA was performed as described in the manufacturer’s guidelines. Briefly, ONH astrocytes and LC cells were plated in 12-well plates containing DMEM with 10% fetal bovine serum. At 30%–40% confluence, transfection of siRNA was performed. In one tube, 4 ul of DharmaFECT 1 Transfection Reagent (T-2001–01; Dharmacon, Lafayette, CO) was mixed gently with 196 μl of Opti-MEM medium (Invitrogen Corporation, Carlsbad, CA) and was incubated for 5 min at room temperature. In separate tubes, various concentrations of siRNAs were mixed gently with 196 μl of Opti-MEM medium. These two tubes were combined, gently mixed, and incubated for 20 min at room temperature. After incubation, Opti-MEM medium was added to obtain a final volume of 2 ml for each well (25 nM and 50 nM of Smad3 siRNA, 100 nM for Smad2 siRNA and 100 nM of non-targeting siRNA controls). Cells were twice washed with sterile PBS and were incubated with siRNA transfection solution for 48 h at 37 °C. Subsequently, cells were washed with serum-free DMEM medium and were treated with TGF-β2 (5 ng/ml) in serum-free DMEM medium for 24 h. The culture medium and cell lysates were analyzed for RSmad2/3, fibronectin, and plasminogen activator inhibitor (PAI)-1.

## Results

### Increased TGF-β2 expression in glaucomatous human ONH tissues

To confirm that TGF-β2 expression is increased in the human glaucomatous ONH, we first examined four age-matched normal and glaucomatous ONH tissues. Figure 1A,C demonstrate TGF-β2 immunostaining (red) merged with glial fibrillary acidic protein (GFAP; green) in a representative ONH region of a normal human donor (97 years). Figure 1B,D represent TGF-β2 immunostaining merged with GFAP in a representative glaucomatous ONH sample (99 years). TGF-β2 was localized in the pre-lamina and LC region along axon bundles, and was also associated with blood vessels. Significantly, TGF-β2 and GFAP staining was higher in the glaucomatous ONH tissues. In addition, there was increased co-localization of TGF-β2 with GFAP in the glaucomatous ONH tissues compared to normal ONH tissues. No staining was observed in negative controls that included normal IgG (not shown) or omission of the primary antibody (Figure 1E,F). The relative intensity of TGF-β2 was measured by ImageJ software (NIH) and indicated that TGF-β2 protein levels were increased significantly in the age-matched glaucomatous ONH tissues compared to the controls (p<0.0013*;* **verses normal human ONH tissues; n=4; Figure 1G).

### Presence of TGF-β2 in ONH cells

**Figure 2:**
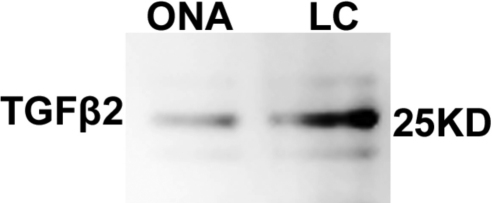
Western blot analysis of transforming growth factor (TGF)-β2 protein in optic nerve head (ONH) cells. ONH astrocytes and lamina cribrosa (LC) cells were grown in serum-free medium for 24 h. Culture medium (20X concentrated) was subjected to western blot analysis for TGF-β2 expression. A positive band for TGF-β2 at 25 kDa was detected in western blots, indicating that ONH astrocytes and LC cells secrete TGF-β2.

To examine the role of elevated TGF-β2 in ECM modulation in the ONH, we next sought to determine whether ONH astrocytes and LC cells secrete endogenous TGF-β2. Confluent ONH astrocytes and LC cells were kept in serum-free medium for 24 h. The conditioned medium (concentrated 20×) was subjected to western blot analysis of TGF-β2. ONH astrocytes and LC cells secreted endogenous TGF-β2 (Figure 2). In addition, endogenous TGF-β2 was present in lysates obtained from human ONH tissues (data not shown), confirming our immunohistochemical results in Figure 1. Recombinant TGF-β2 was used as a positive control for the western blots (data not shown), and this standard, as well as the samples from ONH astrocytes and LC cells, had similarly sized 25 kDa bands (Figure 2). These results confirm that ONH astrocytes and LC cells secrete TGF-β2.

### Recombinant TGF-β2 increases synthesis and deposition of ECM proteins in ONH astrocytes and LC cells

**Figure 3:**
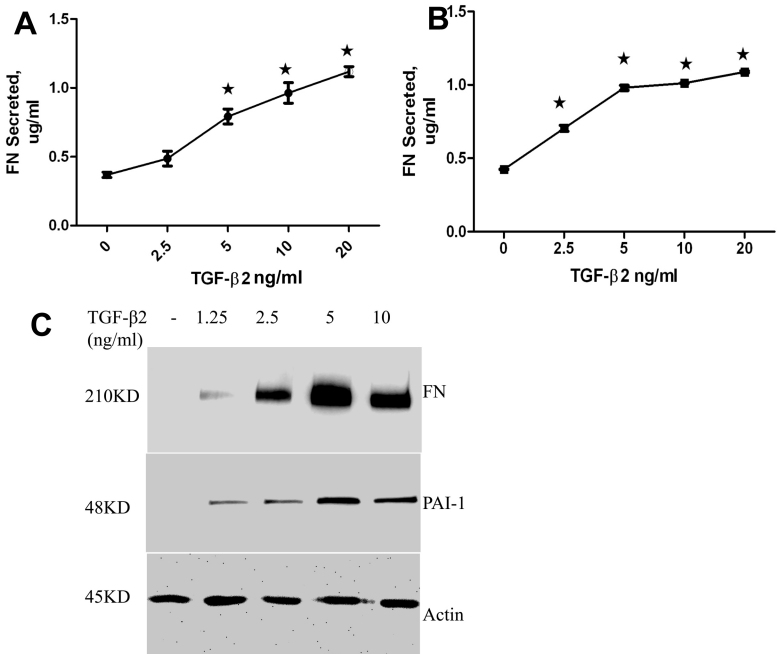
Effect of recombinant transforming growth factor (TGF)-β2 on extracellular matrix (ECM) protein synthesis and deposition in optic nerve head (ONH) astrocytes and lamina cribrosa (LC) cells. Three different ONH astrocytes cell lines and four different LC cell lines were treated with various concentrations of recombinant TGF-β2 (0, 2.5, 5, 10, and 20 ng/ml) for 24 h, and culture medium and cell lysates were subjected to an analysis of ECM proteins. **A**: The effect of TGF-β2 on FN secretion in ONH astrocytes, and **B**: LC cells, was examined using enzyme-linked immunosorbent assay (ELISA). Statistical analysis was performed in Graph Pad Prism using one-way ANOVA (n=3 *, p<0.0001 versus control). Recombinant TGF-β2 increased soluble fibronectin (FN) in a dose dependant fashion. **C**: Cell lysates obtained from LC cells were subjected to western blot analysis of cellular FN and plasminogen activator inhibitor (PAI)-1 in LC cells. Recombinant TGF-β2 increased synthesis of cellular FN and PAI-1 in a dose dependant manner.

To delineate the effect of exogenous TGF-β2 on ECM proteins in vitro, we sought to determine whether the addition of human recombinant TGF-β2 stimulates ECM expression in ONH astrocytes and LC cells. We performed dose response curves for the effects of TGF-β2 on fibronectin and PAI-1 production. Optic nerve head astrocytes (n=3) and LC cells (n=3) were treated with various concentrations of recombinant TGF-β2 (1.25, 2.5, 5, 10, and 20 ng/ml) for 48 h. The effect of TGF-β2 on secreted fibronectin (FN) was examined by ELISA immunoassay (ONH astrocytes - Figure 3A, and LC cells - Figure 3B), and western blot analysis was used to examine cellular FN and PAI-1 (Figure 3C). In the ELISA immunoassay, recombinant TGF-β2 increased soluble FN in a dose dependant manner in both cell types (Figure 3A,B). Recombinant TGF-β2 (5 ng/ml) increased soluble FN levels twofold compared to the vehicle controls. The response of TGF-β2 treatment on FN and PAI-1 protein was measured by western blot analysis (cell lysates) and by ELISA (secretion). The secretion of fibronectin appeared to be dose dependent up to the highest TGF-β2 concentration tested (20 ng/ml). However, the induction of FN and PAI-1 in the cell lysates appeared to reach a maximum at 5 ng/ml, with less induction at 10 ng/ml. This apparent reduction in the TGF-β2 response may be due to enhanced secretion of FN from the cell at the higher dose, which would correlate with the increase in FN secretion seen in the ELISA results. Since a concentration of 5 ng/ml significantly increased soluble FN, we elected to utilize this concentration for subsequent studies.

### Recombinant TGF-β2 activates the canonical Smad signaling pathway in ONH astrocytes and LC cells

**Figure 4:**
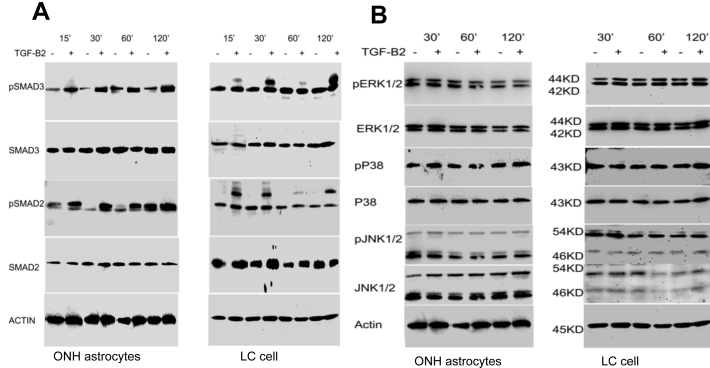
Effect of transforming growth factor (TGF)-β2 on activation of Smad2/3 in optic nerve head (ONH) astrocytes and lamina cribrosa (LC) cells. ONH astrocytes and LC cells were incubated with recombinant TGF-β2 (5 ng/ml) for 0, 15, 30, 60, and 120 min. **A**: Cell lysates were analyzed for phosphorylated Smad3, total Smad3, phosphorylated Smad2, Smad2, and actin. **B**: ONH astrocytes and LC cells were treated with or without TGF-β2 for 30 min, 60 min, and 120 min, and total cell lysates were also examined for phosphorylated extracellular signal-regulated kinases (ERK)1/2, total ERK1/2, phosphorylated p38, total p38, phosphorylated c-Jun N-terminal kinases (JNK)1/2, total JNK1/2, and actin. Recombinant TGF-β2 increased phosphorylation of Smad3 and Smad2 in ONH astrocytes as well as Smad3 phosphorylation in LC cells at 15 min, 30 min, 60 min and 120 min but did not alter phosphorylation ERK, p38, and JNK1/2 in ONH astrocytes and LC cells. These signaling proteins were detected at expected molecular sizes.

To understand the signaling pathways used by TGF-β2 to stimulate ECM proteins, we sought to study whether recombinant TGF-β2 activated Smad and/or non-Smad signaling pathways in isolated ONH astrocytes and LC cells. Since the canonical TGF-β signaling pathway involves activation of Smads via phosphorylation of Smad2 and/or Smad3, we sought to determine whether TGF-β2 phosphorylates Smad2/3 in isolated ONH astrocytes and LC cells. ONH astrocytes and LC cells were incubated with TGF-β2 (5 ng/ml) for 0, 15, 30, 60, and 120 min, and phosphorylation of Smad2 and Smad3 was examined by western immunoblotting. Recombinant TGF-β2 increased the phosphorylation of Smad2 and Smad3 in ONH astrocytes in a time dependent manner, and increased Smad3 phosphorylation in LC cells compared to baseline controls. It appears that TGF-β2 also phosphorylates higher molecular bands for pSmad2 and pSmad3, which are recognized by respective antibodies. Total Smad2, Smad3, and actin levels did not change upon treatment with TGF-β2 (Figure 4A). These findings suggest that recombinant TGF-β2 can activate Smad2/3 in ONH astrocytes and LC cells.

Next, we sought to study whether TGF-β2 also activates non-Smad signaling pathways such as ERK1/2, p38, or JNK1/2 in ONH astrocytes and LC cells. We examined the phosphorylation of these signal kinases using immunoblotting with phospho-specific antibodies (Figure 4B). In ONH astrocytes and LC cells, recombinant TGF-β2 did not alter the phosphorylation of ERK1/2 compared to the baseline control at 15, 30, 60, or 120 min. Similarly, recombinant TGF-β2 did not alter phosphorylation of p38 or JNK1/2 in ONH astrocytes or LC cells. Detection of equal amounts of actin as well as total ERK1/2, p38 or JNK1/2 ensured equal loading of total proteins. Thus, TGF-β2-induced ECM protein expression did not appear to utilize downstream signaling activation of ERK1/2, p38, or JNK1/2 in ONH astrocytes and LC cells.

### TGF-β2 increases co-localization of phosphorylated Smad3 and co-Smad4 in LC cells

**Figure 5:**
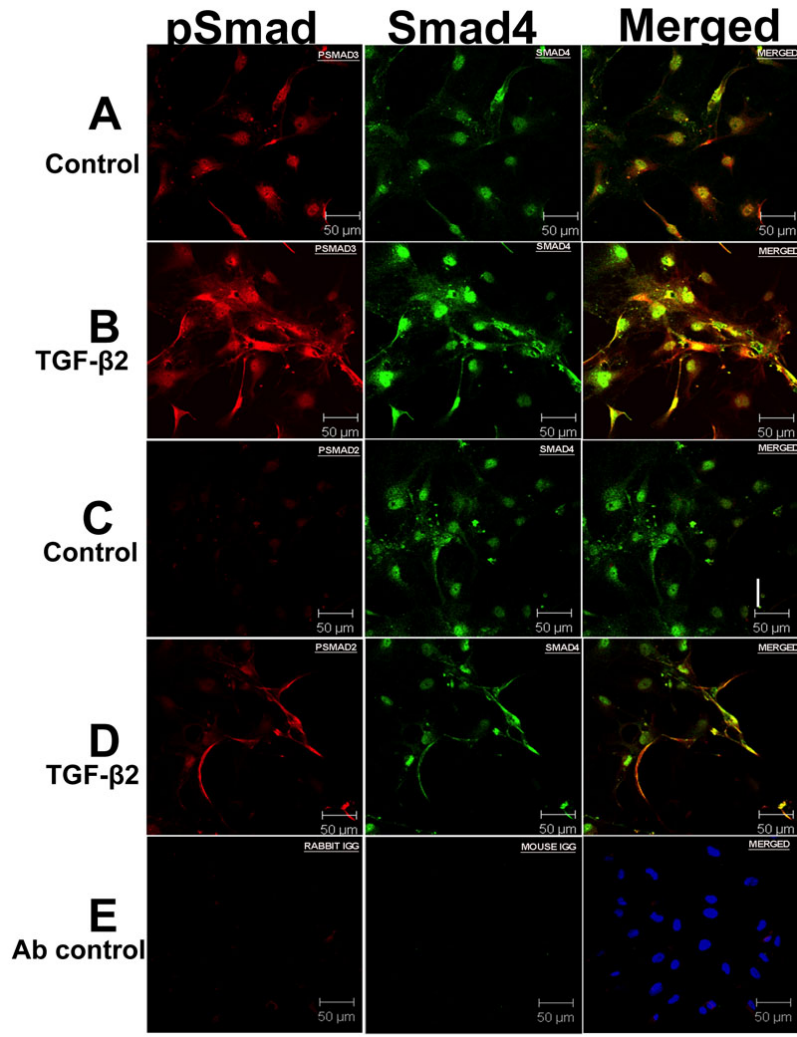
Effect of recombinant transforming growth factor (TGF)-β2 on localization of Smad2/3 with Co-Smad4 in lamina cribrosa (LC) cells. LC cells were incubated with recombinant TGF-β2 (5 ng/ml) for 60 min. Cells were then fixed with 3.5% formaldehyde solution and were stained with antibodies for pSmad3, pSmad2, and Smad4. **A**: Co-localization of pSmad3 (red) and Smad4 (green) in untreated LC cells, and **B**: in TGF-β2 treated cells. **C**: Co-localization of pSmad2 (red) and Smad4 (green) in untreated LC cells, and **D**: in TGF-β2 treated cells. **E**: Negative controls consisted of LC cells incubated with rabbit and mouse IgG and were co-stained with nuclear stain DAPI (blue).

To further study the canonical Smad signaling pathway in LC cells, we performed co-localization of pSmads with Co-Smad4. Activated receptor Smads form a complex with Co-Smad4, which facilitates nuclear import and interaction with the target genes. Since our previous experiments demonstrated that recombinant TGF-β2 phosphorylated Smad2/3, we sought to examine whether TGF-β2 increases the co-localization of pSmad 2 or pSmad3 with Co-Smad4 in LC cells (Figure 5). Even in serum deprived, untreated LC cells, there was some co-localization of pSmad3 with Co-Smad4 in the nucleus (Figure 5A), indicating the presence of an endogenous autocrine TGF-β signaling pathway via Smad3. In contrast, there is no detectable level of immunostaining for pSmad2 in untreated LC cells (Figure 5C). However, in TGF-β2 stimulated LC cells, the co-localization of phosphorylated Smad3 and Co-Smad4 was increased in both the cytoplasm and nucleus (Figure 5B). In addition, p-Smad2 and Co-Smad4 levels were increased and these factors were co-localized in TGF-β2 stimulated LC cells (Figure 5D). Similar findings were found in the ONH astrocytes (data not shown). These findings support our previous immunoblotting results indicating that TGF-β2 activates Smads phosphorylation, which then translocates the Smad2/3/4 complex to the nucleus.

### Inhibition of the type I TGF-β receptor activity or inhibition of Smad3 phosphorylation blocks TGF-β2 stimulation of ECM proteins

**Figure 6:**
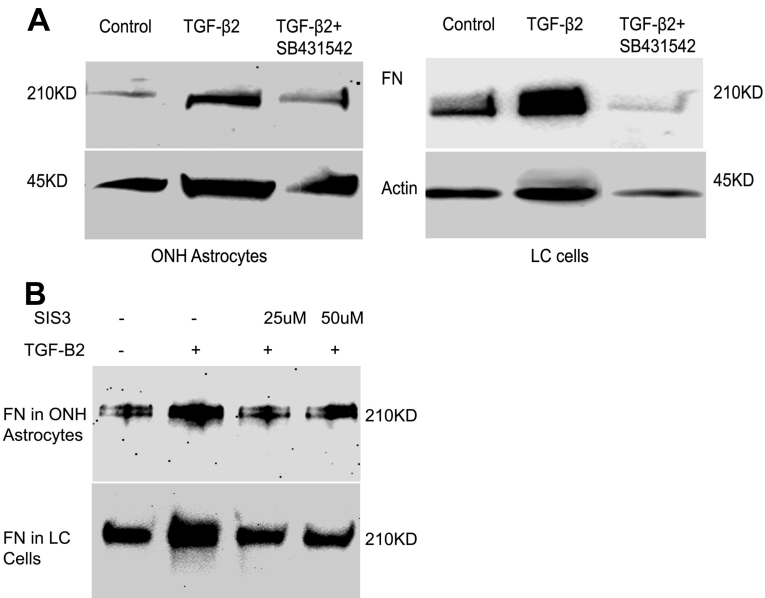
Effect of inhibition of transforming growth factor (TGF)-β receptor I or Smad3 phosphorylation on TGF-β2-driven extracellular matrix (ECM) stimulation. Optic nerve head (ONH) astrocytes and lamina cribrosa (LC) cells were pre-incubated with a type I TGF-β receptor inhibitor (SB431542, 10 uM) or an inhibitor of Smad3 phosphorylation (SIS3, 25 and 50 uM), 1 h before treatment with recombinant TGF-β2 (5 ng/ml) for 24 h. **A**: Total cell lysates, and **B**: the conditioned medium were subjected to western blot analysis of fibronectin. **A**: The effect of SB431542 on TGF-β2 stimulated cellular fibronectin (FN) was assessed by western blot in ONH astrocytes and LC cells. **B**: Effect of inhibition of phosphorylation of Smad3 on TGF-β2 stimulated ECM was examined by western blot analysis of secreted FN in ONH astrocytes and LC cells. Inhibition of Smad3 phosphorylation reduced TGF-β2 stimulated FN secretion in ONH astrocytes and LC cells.

We next sought to examine whether TGF-β2 induced Smad signaling is required for ECM stimulation in ONH astrocytes and LC cells. In the canonical TGF-β2 signaling pathway, secreted TGF-β2 binds to the type II TGF-β receptor, which then activates the type I TGF-β receptor. Activation of type I TGF-β receptor leads to phosphorylation of downstream signaling Smads or non-Smad signaling mediators [40]. We examined the effect of the type I TGF-β receptor inhibitor SB431542, a selective and potent inhibitor of activity of the TGF-β1 activin receptor-like kinases (ALK5). The addition of SB431542 (10 μM) blocked TGF-β2 stimulation of cellular FN in ONH astrocytes and LC cells (Figure 6A). These findings demonstrate that TGF-β2-driven stimulation of ECM proteins requires active TGF-β RI in ONH astrocytes and LC cells.

To elucidate whether TGF-β2-driven stimulation of ECM proteins is mediated via activation of Smad3, we inhibited Smad3 phosphorylation with SIS3, a specific inhibitor of Smad3. ONH astrocytes and LC cells were pre-incubated with SIS3 (25 uM and 50 uM) 1 h before treatment with TGF-β2. We observed that TGF-β2 stimulation of FN secretion in ONH astrocytes and LC cells was reduced to a baseline control by SIS3 treatment (Figure 6B). This finding suggests that TGF-β2-driven stimulation of ECM proteins requires activation of Smad3.

### Effect of SB431542 and SIS3 on TGF-β2-Stimulated Smad Signaling

**Figure 7:**
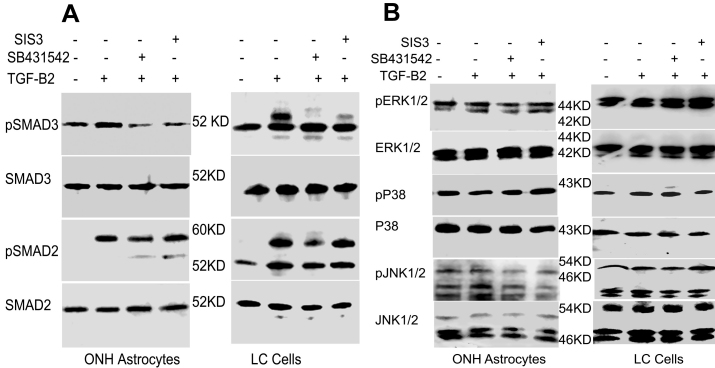
Effect of inhibition of transforming growth factor (TGF)-β receptor I or Smad3 phosphorylation on Smad and non-Smad signaling pathways. Optic nerve head (ONH) astrocytes and lamina cribrosa (LC) cells were pre-incubated with SB431542 (10 uM) or SIS3 (25 uM), 1h before treatment with TGF-β2. ONH astrocytes and LC cells were then treated with recombinant TGF-β2 for 1 h and total cell lysates were subjected to western blot analysis for phosphorylation of Smad and non-Smad signaling pathways. **A**: western blot analysis of pSmad3, Smad3, pSmad2, and Smad2 in ONH astrocytes and LC cells. **B**: western blot analysis of phosphorylated extracellular signal-regulated kinases (ERK)1/2, total ERK1/2, phosphorylated p38, total p38, phosphorylated c-Jun N-terminal kinases (JNK)1/2, and total JNK1/2 in ONH astrocytes and LC cells. Samples were run in the same gel but not in the order presented. SB431542 inhibited TGF-β2 induced phosphorylation of Smad2/3, whereas SIS3 selectively inhibited phosphorylation of Smad3. The inhibitors had no effect on members of the non-Smad pathway.

Next, we examined whether the effects of the TGFβR1 (SB431542) or Smad 3 (SIS3) inhibitors on TGF-β2 stimulated ECM were mediated via inhibition of TGF-β2 induced signaling. We pre-incubated ONH astrocytes and LC cells with either SB431542 or SIS3 for 1 h and then treated them with TGF-β2 for 1 h. Total cell lysates were subjected to an analysis of phosphorylation of Smad and non-Smad signaling molecules. TGF-β2 alone caused increased phosphorylation of Smad2 and Smad3 after 1 h of treatment (Figure 7A). The addition of SB431542 blocked phosphorylation of Smad2 and Smad3, indicating inhibition of the downstream signaling pathway through TGF-βR-I. Total protein levels of Smad2 and Smad3 did not change with TGF-β2 treatment alone or with the combination of TGF-β2 and SB431542, compared to the vehicle control (Figure 7A). In addition, TGF-β2 alone or TGF-β2 and SB431542 did not alter phosphorylation of ERK1/2, p38 or JNK1/2 (Figure 7B), further supporting our previous findings that TGF-β2 does not activate non-Smad signaling pathways in ONH astrocytes or LC cells. SIS3 selectively inhibited TGF-β2 induced phosphorylation of Smad3 without affecting total Smad3, and SIS3 did not inhibit phosphorylation of Smad2 levels in ONH astrocytes and LC cells (Figure 7A). Moreover, SIS3 did not affect phosphorylation of non-Smad signaling molecules such as ERK1/2, p38, or JNK1/2 (Figure 7B).

### siRNA knockdown of Smad3 or Smad2 blocks TGF-β2-driven stimulation of FN and PAI-1 in ONH astrocytes and LC Cells

**Figure 8:**
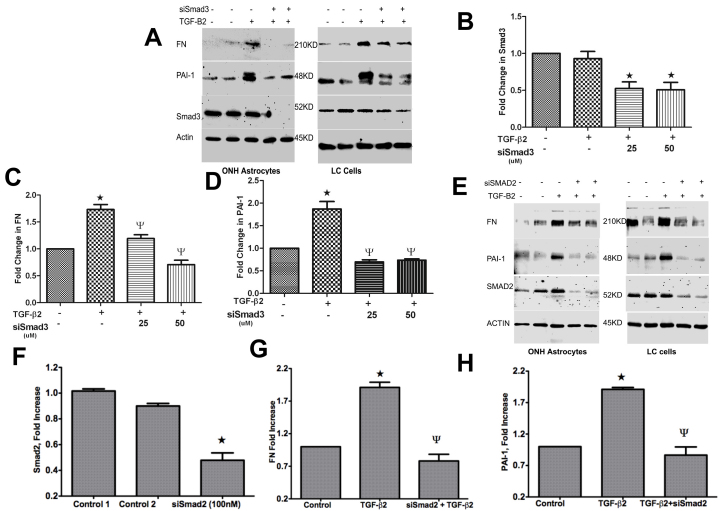
Effect of Smad2 or Smad3 siRNA on transforming growth factor (TGF)-β2 stimulation of fibronectin (FN) and plasminogen activator inhibitor (PAI)-1 in optic nerve head (ONH) astrocytes and lamina cribrosa (LC) cells. **A**: ONH astrocytes and LC cells were treated with small interfering RNA (siRNA) controls (lane 1=Non-targeting siRNA; lane 2=RISC-Free siRNA) or with Smad3 siRNA (25 nM and 50 nM; lanes 4–5) for 48 h, and were then treated with recombinant TGF-β2 (5 ng/ml) for 24 h. Cellular FN, PAI-1, total Smad3, and actin were assessed by western blot. **B**: Relative densities of Smad3 and actin in ONH astrocytes were measured using densitometric analysis of the western blots. Smad3 was normalized to actin and the fold change in Smad3 over the vehicle control was plotted (n=3,* p<0.001 versus control). **C** and **D**: Relative FN or PAI-1 was normalized to actin and the fold change in FN (**C**) or PAI-1(**D**) over the vehicle control was plotted (n=3,* p<0.001 verses TGF-β2 treated). **E**: ONH astrocytes and LC cells were treated with siRNA controls (lane 1=Non-targeting siRNA; lane 2=RISC-Free siRNA) or with Smad2 siRNA (lanes 4–5, 100 nM in duplicate) for 48 h, and were then treated with recombinant TGF-β2 (5 ng/ml) for 24 h. **F**: Cellular FN, PAI-1, total Smad2, and actin were assessed by western blot. Smad2 levels in ONH astrocytes were normalized to actin and the fold change in Smad2 over the vehicle control was plotted (n=3, *p<0.0018 verses control). **G** and **H**: Relative densities of FN, PAI-1, and actin were measured using densitometric analysis. Relative densities of FN or PAI-1 were normalized to actin and the fold change in FN (**G**) or PAI-1(**H**) over vehicle the control was plotted (n=3, p<0.0048 for FN and p<0.0015 for PAI-1, * verses TGF-β2 treated).

Our previous pharmacological experiments suggested that Smad3 is required for TGF-β2 stimulation of ECM proteins. In addition to Smad3, TGF-β2 also activated Smad2 phosphorylation in ONH astrocytes and LC cells (Figure 4 and Figure 5), indicating the potential role of Smad2 in TGF-β2 signaling in ONH astrocytes and LC cells. To examine whether Smad2 or Smad3 are preferentially used by TGF-β2 to induce ECM stimulation, we conducted siRNA knockdown of Smad3 (Figure 8A-D) and Smad2 (Figure 8E-H) along with control siRNAs (RISC-Free siRNAs and non-targeting siRNAs). The cells were treated with siRNAs for 48 h and were then treated with TGF-β2 for 24 h. Fibronectin, PAI-1, Smad3, Smad2, and actin protein levels were assessed by western immunoblotting and were measured by densitometric analysis. Transfection with Smad3-siRNA (25 nM and 50 nM) resulted in a significant (p<0.001) reduction of Smad3 protein levels compared to control siRNAs (50 nM; Figure 8A,B). Control siRNAs did not significantly alter Smad3 protein levels compared to the vehicle control, indicating sequence-specific silencing of our target siRNAs. Next, ONH astrocytes and LC cells were treated with TGF-β2 with and without siRNAs for Smad3. Western blot and densitometric analysis demonstrated that reduction of Smad3 via siRNA (25 nM and 50 nM) significantly blocked the stimulatory effects of TGF-β2 on FN and PAI-1 proteins (Figure 8A,C,D). These results indicate that Smad3 is required for TGF-β2 stimulation of ECM proteins in ONH astrocytes and LC cells.

Next, we examined whether TGF-β2 requires Smad2 to induce ECM stimulation in ONH astrocytes and LC cells. Fibronectin, PAI-1, Smad2, and actin protein levels were assessed by western blot (Figure 8E) and were measured by densitometric analysis (Figure 8F-H). Transfection with Smad2 siRNA (100 nM) resulted in significant (n=3, *p<0.0018 verses control*)* reduction of Smad2 protein levels compared to control siRNAs (100nM) in ONH astrocytes and LC cells (Figure 8E,F). Control siRNAs did not significantly alter Smad2 protein levels compared to the vehicle control, indicating sequence-specific silencing of our target siRNAs. Western blot and densitometric analysis demonstrated that reduction of Smad2 via siRNA significantly blocked the stimulatory effects of TGF-β2 on FN and PAI-1 proteins (Figure 8E,F-H). These results indicate that both Smad3 and Smad2 are downstream signaling proteins used for TGF-β2 stimulation of ECM proteins in ONH astrocytes and LC cells.

## Discussion

In glaucoma, the LC region of the ONH is the primary site of injury that leads to the death of RGC axons [44]. The LC is the weakest part of the ocular globe and is thus more vulnerable to elevated IOP. Chronic IOP elevation is thought to cause cupping and excavation of the optic disc, collapse and remodeling of the LC, and activation of ONH astrocytes [4,10,45]. Alteration in the synthesis, deposition, and quality of ECM proteins in the LC region has been associated with glaucomatous ONH changes and is thought to be detrimental to RGC axons. For example, remodeling of the ECM in the glaucomatous ONH may contribute to the backward bowing of the laminar plates and compression of RGC axons. Remodeling of the ECM includes changes in fibrillar collagens, basement membrane components, and degradation of elastin fibers [21]. Extracellular matrix changes previously reported in the glaucomatous ONH include (a) increased amounts of collagens I, IV, and VI [21], and (b) elastin fiber degradation [20]. Thus, the alteration of ECM proteins in the LC region may disrupt nutritional and mechanical support to RGC axons, resulting in RGC atrophy.

Transforming growth factor-β2 is a known fibrotic modulator. Pena and colleagues reported increased immunohistochemical expression of TGF-β2 in the glaucomatous ONH [38]. Our results are the first to independently verify this observation. However, Pena et al., (1999) did not elucidate the cellular source of TGF-β2 expression pattern in the glaucomatous ONH. There are at least five different cell types reported to be present within the human ONH, including astrocytes, LC cells, microglia, endothelial cells, and pericytes [9,15,46,47]. Many studies assume that ONH astrocytes and LC cells respond to elevated IOP by increasing TGF-β2 synthesis and secretion, which in turn causes alteration of ECM protein expression. However, definitive studies verifying this assumption were lacking.

Consistent with the Pena study, we observed that TGF-β2 is elevated in the glaucomatous ONH. Interestingly, TGF-β2 was significantly increased in the LC region of the glaucomatous ONH, and TGF-β2 co-localized with GFAP positive cells, indicating that ONH astrocytes may be a major source of TGF-β2 in vivo. We also have demonstrated that treatment of ONH astrocytes and LC cells with TGF-β2 increased ECM protein synthesis in both ONH astrocytes and LC cells, suggesting that both ONH astrocytes and LC are capable of responding to TGF-β2 in vivo. This is the first report to illustrate how human LC cells respond to TGF-β2 with increased ECM protein synthesis and secretion.

TGF-β2 may alter ECM metabolism via several mechanisms. Treatment of ONH astrocytes and LC cells with recombinant TGF-β2 increased soluble FN and PAI-1 in a dose dependant manner. PAI-1 is involved in fibrosis by regulating the activity of matrix metalloproteases (MMPs), and matrix metalloproteases have been shown to be involved in ECM remodeling of the glaucomatous ONH [48,49]. Neumann et al. demonstrated that recombinant TGF-β2 increases MMP-2 and PAI-1 in ONH astrocytes [50]. Increased type I and VI collagen and elastin are thought to alter the mechanical and elastic properties of LC in glaucomatous ONH. Fuchshofer and colleagues [17] showed that TGF-β2 increases FN, collagen I and IV, tissue transglutaminase, and CTGF in ONH astrocytes. Results from the present study confirm the findings of these previous studies, showing that recombinant TGF-β2 increases FN, PAI-1, elastin, and collagen I and collagen VI in ONH astrocytes. However, in addition to ONH astrocytes, our current study demonstrated that LC cells also respond to recombinant TGF-β2 by increasing FN, PAI-1, elastin, and collagen I, and VI. Since LC cells secrete TGF-β2 and respond to recombinant TGF-β2 via increasing ECM proteins, it is possible that LC cells may also play an important role in altering the mechanical and elastic properties of LC. These observations reinforce the hypothesis that TGF-β2 is involved in ECM changes in the glaucomatous ONH.

The signaling pathway used by TGF-β2 to increase ECM expression in human ONH astrocytes and LC cells has not been previously identified. In many cell types, TGF-β2 induced fibrosis utilizes the canonical Smad pathway via Smad2 and Smad3 [51]. Importantly, in some cell types, non-Smad pathways, including ERK, p38MAPK, and JNK, have also been reported to be activated by TGF-β2 [40]. Thus, determining which pathway(s) ONH astrocytes and LC cells utilize to regulate ECM protein synthesis and secretion is of great importance with respect to the pathophysiology of glaucoma.

Our results demonstrated the presence of TGF-β2, endogenous pSmad2/3 and their co-localization with Co-Smad4, thus indicating that ONH astrocytes and LC cells possess autocrine TGF-β2 mediated Smad signaling. In addition, treatment with exogenous TGF-β2, increased pSmad2 and pSmad3 levels and their co-localization with Co-Smad4 in the nucleus, indicating that isolated ONH astrocytes and LC cells can also respond to exogenous TGF-β2 via activation of canonical Smad signaling. Interestingly, TGF-β2 did not activate phosphorylation of non-Smad signaling pathways such as ERK1/2, p38, or JNK1/2 in either ONH astrocytes or LC cells. Therefore, TGF-β2 does not appear to stimulate non-Smad pathways in ONH astrocytes and LC cells.

We next determined if the canonical Smad signaling pathway was required for TGF-β2-driven ECM protein regulation. Pre-incubation with SB431542 or SIS3 reversed TGF-β2 stimulated FN and PAI-1 expression to vehicle control levels. SB431532 is a potent and selective inhibitor of TGF-ßRI activity. SB431542 has been reported to inhibit pro-fibrotic actions of TGF-β2 in skin fibroblasts and hepatic cells. SB431542 inhibited TGF-β2 induced phosphorylation of Smad2 and Smad3 without altering total Smad2 or Smad3 protein levels. Furthermore, the Smad3 inhibitor SIS3 [52] reduced TGF-β2 regulated phosphorylation of Smad3 but not Smad2. As expected, neither SB431542 nor SIS3 had an effect on the non-Smad signaling pathways assessed by examining the phosphorylation of ERK1/2, p38, and JNK1/2.

We further examined the effect Smad2 and Smad3 knockdown on TGF-β2 stimulated ECM protein expression in ONH astrocytes and LC cells. siRNA knockdown of Smad2 and Smad3 reduced the total amount of Smad2 and Smad3 in ONH astrocytes and LC cells. Knockdown of Smad2 or Smad3 inhibited TGF-β2 stimulation of FN and PAI-1 in ONH astrocytes and LC cells. Therefore, Smad2 as well as Smad3 is used for TGF-β2 stimulated ECM proteins. Since knockdown of either Smad2 or Smad3 completely reversed TGF-β2 stimulated ECM proteins to control levels, both signaling molecules may be required for TGF-β2 stimulation of ECM proteins.

### Conclusions

The present study provides both in vivo and in vitro evidence to support the conclusion that TGF-β2 is involved in ECM remodeling by cells of the human ONH. In addition, TGF-β2-driven ECM stimulation requires activation of the canonical Smad signaling pathway via Smad2/3. Non-Smad signaling pathways do not appear to be involved in TGF-β2 stimulation of ECM protein synthesis and secretion by ONH astrocytes or LC cells. Inhibition of the type I TGF-β receptor or knockdown of either Smad2 or Smad3 reversed TGF-β2 stimulated ECM proteins in ONH astrocytes and LC cells. Therefore, inhibition of these downstream signals may provide a therapeutic target to prevent ECM remodeling in the glaucomatous ONH.

## References

[1] Quigley HA (1999). Neuronal death in glaucoma.. Prog Retin Eye Res.

[2] Rohen JW (1983). Why is intraocular pressure elevated in chronic simple glaucoma? Anatomical considerations.. Ophthalmology.

[3] Sommer A, Katz J, Quigley HA, Gottsch JD, Javitt J, Singh K (1991). Relationship between intraocular pressure and primary open angle glaucoma among white and black Americans. The Baltimore Eye Survey.. Arch Ophthalmol.

[4] Hernandez MR, Pena JD (1997). The optic nerve head in glaucomatous optic neuropathy.. Arch Ophthalmol.

[5] Levy NS, Crapps EE (1984). Displacement of optic nerve head in response to short-term intraocular pressure elevation in human eyes.. Arch Ophthalmol.

[6] Anderson DR (1969). Ultrastructure of human and monkey lamina cribrosa and optic nerve head.. Arch Ophthalmol.

[7] Emery JM, Landis D, Paton D, Boniuk M, Craig JM (1974). The lamina cribrosa in normal and glaucomatous human eyes.. Trans Am Acad Ophthalmol Otolaryngol.

[8] Quigley HA, Hohman RM, Addicks EM, Massof RW, Green WR (1983). Morphologic changes in the lamina cribrosa correlated with neural loss in open-angle glaucoma.. Am J Ophthalmol.

[9] Hernandez MR (2000). The optic nerve head in glaucoma: role of astrocytes in tissue remodeling.. Prog Retin Eye Res.

[10] Quigley HAAE (1981). Regional differences in the structure of the lamina cribrosa and their relation to glaucomatous optic nerve damage.. Arch Ophthalmol.

[11] Oyama T, Abe H, Ushiki T (2006). The connective tissue and glial framework in the optic nerve head of the normal human eye: light and scanning electron microscopic studies.. Arch Histol Cytol.

[12] Birch M, Brotchie D, Roberts N, Grierson I (1997). The three-dimensional structure of the connective tissue in the lamina cribrosa of the human optic nerve head.. Ophthalmologica.

[13] Albon J, Purslow PP, Karwatowski WS, Easty DL (2000). Age related compliance of the lamina cribrosa in human eyes.. Br J Ophthalmol.

[14] Kirwan RP, Fenerty CH, Crean J, Wordinger RJ, Clark AF, O'Brien CJ (2005). Influence of cyclical mechanical strain on extracellular matrix gene expression in human lamina cribrosa cells in vitro.. Mol Vis.

[15] Hernandez MR, Igoe F, Neufeld AH (1988). Cell culture of the human lamina cribrosa.. Invest Ophthalmol Vis Sci.

[16] Lambert W, Agarwal R, Howe W, Clark AF, Wordinger RJ (2001). Neurotrophin and neurotrophin receptor expression by cells of the human lamina cribrosa.. Invest Ophthalmol Vis Sci.

[17] Fuchshofer R, Birke M, Welge-Lussen U, Kook D, Lutjen-Drecoll E (2005). Transforming growth factor-beta 2 modulated extracellular matrix component expression in cultured human optic nerve head astrocytes.. Invest Ophthalmol Vis Sci.

[18] Zode GS, Clark AF, Wordinger RJ (2007). Activation of the BMP canonical signaling pathway in human optic nerve head tissue and isolated optic nerve head astrocytes and lamina cribrosa cells.. Invest Ophthalmol Vis Sci.

[19] Zode GS, Clark AF, Wordinger RJ (2009). Bone morphogenetic protein 4 inhibits TGF-beta2 stimulation of extracellular matrix proteins in optic nerve head cells: role of gremlin in ECM modulation.. Glia.

[20] Hernandez MR (1992). Ultrastructural immunocytochemical analysis of elastin in the human lamina cribrosa. Changes in elastic fibers in primary open-angle glaucoma.. Invest Ophthalmol Vis Sci.

[21] Hernandez MR, Andrzejewska WM, Neufeld AH (1990). Changes in the extracellular matrix of the human optic nerve head in primary open-angle glaucoma.. Am J Ophthalmol.

[22] Hernandez MR, Ye H (1993). Glaucoma: changes in extracellular matrix in the optic nerve head.. Ann Med.

[23] Morrison JC, Dorman-Pease ME, Dunkelberger GR, Quigley HA (1990). Optic nerve head extracellular matrix in primary optic atrophy and experimental glaucoma.. Arch Ophthalmol.

[24] Pena JD, Agapova O, Gabelt BT, Levin LA, Lucarelli MJ, Kaufman PL, Hernandez MR (2001). Increased elastin expression in astrocytes of the lamina cribrosa in response to elevated intraocular pressure.. Invest Ophthalmol Vis Sci.

[25] Johnson EC, Jia L, Cepurna WO, Doser TA, Morrison JC (2007). Global changes in optic nerve head gene expression after exposure to elevated intraocular pressure in a rat glaucoma model.. Invest Ophthalmol Vis Sci.

[26] Kirwan RP, Crean JK, Fenerty CH, Clark AF, O'Brien CJ (2004). Effect of cyclical mechanical stretch and exogenous transforming growth factor-beta1 on matrix metalloproteinase-2 activity in lamina cribrosa cells from the human optic nerve head.. J Glaucoma.

[27] Fukuchi T, Ueda J, Hanyu T, Abe H, Sawaguchi S (2001). Distribution and expression of transforming growth factor-beta and platelet-derived growth factor in the normal and glaucomatous monkey optic nerve heads.. Jpn J Ophthalmol.

[28] Verrecchia F, Mauviel A (2007). Transforming growth factor-beta and fibrosis.. World J Gastroenterol.

[29] Schnaper HW, Hayashida T, Hubchak SC, Poncelet AC (2003). TGF-beta signal transduction and mesangial cell fibrogenesis.. Am J Physiol Renal Physiol.

[30] Yu L, Border WA, Huang Y, Noble NA (2003). TGF-beta isoforms in renal fibrogenesis.. Kidney Int.

[31] Tripathi RC, Li J, Chan WF, Tripathi BJ (1994). Aqueous humor in glaucomatous eyes contains an increased level of TGF-beta 2.. Exp Eye Res.

[32] Fleenor DL, Shepard AR, Hellberg PE, Jacobson N, Pang IH, Clark AF (2006). TGFbeta2-induced changes in human trabecular meshwork: implications for intraocular pressure.. Invest Ophthalmol Vis Sci.

[33] Fuchshofer R, Welge-Lussen U, Tamm ER (2007). Bone morphogenetic protein-7 is an antagonist of transforming growth factor-beta2 in human trabecular meshwork cells.. Invest Ophthalmol Vis Sci.

[34] Wordinger RJ, Fleenor DL, Hellberg PE, Pang IH, Tovar TO, Zode GS, Fuller JA, Clark AF (2007). Effects of TGF-beta2, BMP-4, and gremlin in the trabecular meshwork: implications for glaucoma.. Invest Ophthalmol Vis Sci.

[35] Gottanka J, Chan D, Eichhorn M, Lutjen-Drecoll E, Ethier CR (2004). Effects of TGF-beta2 in perfused human eyes.. Invest Ophthalmol Vis Sci.

[36] Shepard AR, Millar JC, Pang IH, Jacobson N, Wang WH, Clark AF (2010). Adenoviral gene transfer of active human transforming growth factor-{beta}2 elevates intraocular pressure and reduces outflow facility in rodent eyes.. Invest Ophthalmol Vis Sci.

[37] Robertson JV, Golesic E, Gauldie J, West-Mays JA (2010). Ocular gene transfer of active TGF-beta induces changes in anterior segment morphology and elevated IOP in rats.. Invest Ophthalmol Vis Sci.

[38] Pena JD, Taylor AW, Ricard CS, Vidal I, Hernandez MR (1999). Transforming growth factor beta isoforms in human optic nerve heads.. Br J Ophthalmol.

[39] Attisano L, Lee-Hoeflich ST (2001). The Smads.. Genome Biol.

[40] Rahimi RA, Leof EB (2007). TGF-beta signaling: a tale of two responses.. J Cell Biochem.

[41] Moustakas A, Heldin CH (2005). Non-Smad TGF-beta signals.. J Cell Sci.

[42] Wang S, Hirschberg R (2004). Bone morphogenetic protein-7 signals opposing transforming growth factor beta in mesangial cells.. J Biol Chem.

[43] Wordinger RJ, Agarwal R, Talati M, Fuller J, Lambert W, Clark AF (2002). Expression of bone morphogenetic proteins (BMP), BMP receptors, and BMP associated proteins in human trabecular meshwork and optic nerve head cells and tissues.. Mol Vis.

[44] Howell GR, Libby RT, Jakobs TC, Smith RS, Phalan FC, Barter JW, Barbay JM, Marchant JK, Mahesh N, Porciatti V, Whitmore AV, Masland RH, John SW (2007). Axons of retinal ganglion cells are insulted in the optic nerve early in DBA/2J glaucoma.. J Cell Biol.

[45] Quigley HA, Addicks EM (1980). Chronic experimental glaucoma in primates. II. Effect of extended intraocular pressure elevation on optic nerve head and axonal transport.. Invest Ophthalmol Vis Sci.

[46] Grieshaber MC, Flammer J (2007). Does the blood-brain barrier play a role in Glaucoma?. Surv Ophthalmol.

[47] Yuan L, Neufeld AH (2001). Activated microglia in the human glaucomatous optic nerve head.. J Neurosci Res.

[48] Agapova OA, Ricard CS, Salvador-Silva M, Hernandez MR (2001). Expression of matrix metalloproteinases and tissue inhibitors of metalloproteinases in human optic nerve head astrocytes.. Glia.

[49] Yan X, Tezel G, Wax MB, Edward DP (2000). Matrix metalloproteinases and tumor necrosis factor alpha in glaucomatous optic nerve head.. Arch Ophthalmol.

[50] Neumann C, Yu A, Welge-Lussen U, Lutjen-Drecoll E, Birke M (2008). The effect of TGF-beta2 on elastin, type VI collagen, and components of the proteolytic degradation system in human optic nerve astrocytes.. Invest Ophthalmol Vis Sci.

[51] von Bubnoff A, Cho KW (2001). Intracellular BMP signaling regulation in vertebrates: pathway or network?. Dev Biol.

[52] Jinnin M, Ihn H, Tamaki K (2006). Characterization of SIS3, a novel specific inhibitor of Smad3, and its effect on transforming growth factor-beta1-induced extracellular matrix expression.. Mol Pharmacol.

